# *MMP14* expression and collagen remodelling support uterine leiomyosarcoma aggressiveness

**DOI:** 10.1002/1878-0261.13440

**Published:** 2023-04-28

**Authors:** Jordi Gonzalez‐Molina, Paula Hahn, Raul Maia Falcão, Okan Gultekin, Georgia Kokaraki, Valentina Zanfagnin, Tirzah Braz Petta, Kaisa Lehti, Joseph W. Carlson

**Affiliations:** ^1^ Department of Microbiology, Tumor and Cell Biology Karolinska Institutet Stockholm Sweden; ^2^ Department of Oncology‐Pathology Karolinska Institutet Stockholm Sweden; ^3^ Keck School of Medicine University of Southern California Los Angeles CA USA; ^4^ Department of Cellular Biology and Genetics Federal University of Rio Grande do Norte Natal Brazil; ^5^ Department of Biomedical Laboratory Science Norwegian University of Science and Technology Trondheim Norway

**Keywords:** collagen, extracellular matrix, MMP14, uterine leiomyoma, uterine leiomyosarcoma, YAP

## Abstract

Fibrillar collagen deposition, stiffness and downstream signalling support the development of leiomyomas (LMs), common benign mesenchymal tumours of the uterus, and are associated with aggressiveness in multiple carcinomas. Compared with epithelial carcinomas, however, the impact of fibrillar collagens on malignant mesenchymal tumours, including uterine leiomyosarcoma (uLMS), remains elusive. In this study, we analyse the network morphology and density of fibrillar collagens combined with the gene expression within uLMS, LM and normal myometrium (MM). We find that, in contrast to LM, uLMS tumours present low collagen density and increased expression of collagen‐remodelling genes, features associated with tumour aggressiveness. Using collagen‐based 3D matrices, we show that matrix metalloproteinase‐14 (MMP14), a central protein with collagen‐remodelling functions that is particularly overexpressed in uLMS, supports uLMS cell proliferation. In addition, we find that, unlike MM and LM cells, uLMS proliferation and migration are less sensitive to changes in collagen substrate stiffness. We demonstrate that uLMS cell growth in low‐stiffness substrates is sustained by an enhanced basal yes‐associated protein 1 (YAP) activity. Altogether, our results indicate that uLMS cells acquire increased collagen remodelling capabilities and are adapted to grow and migrate in low collagen and soft microenvironments. These results further suggest that matrix remodelling and YAP are potential therapeutic targets for this deadly disease.

## Graphical abstract

The authors show that, in contrast to benign mesenchymal uterine tumours, uterine leiomyosarcomas are characterised by a low‐density collagenous matrix, which is associated with poor patient prognosis. Molecularly, this is supported by enhanced *MMP14* expression in cancer cells and their low dependence on ECM adhesion to proliferate and migrate.

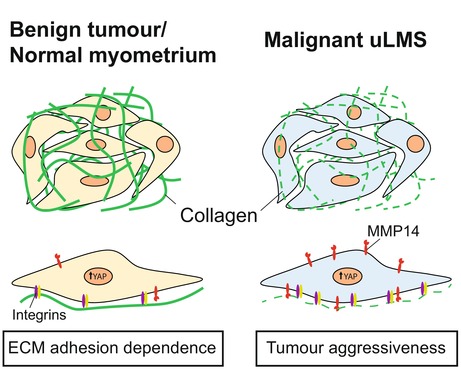

**Abbreviations**
DDR: discoidin domain receptor
ECM: extracellular matrix
EdU: 5‐Ethynyl‐2′‐deoxyuridine
HDM: high‐density matrix
HGU: hyphal growth unit
LG‐ESS: low‐grade endometrial stromal sarcoma
LM: Leiomyoma
MM: normal myometrium
MMP14: atrix metalloproteinase‐14
STS: soft tissue sarcoma
uLMS: uterine leiomyosarcoma
YAP: yes‐associated protein 1

## 1 Introduction

Leiomyosarcomas (LMS) are malignant mesenchymal neoplasms showing features of smooth muscle lineage that represent 10–20% of all newly diagnosed soft tissue sarcomas (STSs) [1]. Uterine LMS (uLMS) are the most common subtype of uterine sarcoma. These tumours are generally treated surgically. Despite apparent complete resection of uLMS tumours, the risk of recurrence is between 50% and 70% [2]. The symptoms of uLMS are similar to those of uterine leiomyomas (LM), common benign fibrotic tumours of the uterus, and thus, most uLMS are diagnosed postoperatively [3, 4]. Treatment of uterine LM includes tissue morcellation, which can lead to the spreading of uLMS in case of misdiagnosis [5, 6]. Understanding the biological basis of uLMS development and progression and its differences with LM is fundamental to improve the diagnosis and treatment of this deadly disease.

The desmoplastic reaction is a characteristic increase in extracellular matrix (ECM) deposition and crosslinking, particularly of fibrillar collagens, that occurs in various types of carcinoma [7]. Similarly, LM are characterised by an enrichment in fibrillar collagens type I and III [8, 9]. The increased deposition, crosslinking and reorganisation of fibrillar collagens causes the stiffening of the ECM and the bulk tissue [10]. Increased tissue stiffness is a characteristic of desmoplastic tumours. Likewise, LM tumours present a higher average stiffness (18.6 kPa) than normal myometrium (MM; 4.9 kPa) [11, 12]. However, changes in fibrillar collagens and tumour stiffness in uLMS have not been systematically studied.

Cells adhere to fibrillar collagens through receptors at the plasma membrane including integrins and discoidin domain receptors (DDRs) [13]. The adhesion of integrins and their stabilisation to form mechanochemical signalling hubs, known as focal adhesions, are favoured in stiff substrates [14]. Adhesion to fibrillar collagens and integrin signalling are involved in the activation of the mechanosensors and mechanotransducers YAP and TAZ [15, 16]. In cancer cells, integrin signalling and YAP/TAZ induce proliferation, stemness, chemoresistance and metastasis [13, 17]. Indeed, YAP is frequently active in LM and STSs and is involved in STS tumorigenesis [18, 19, 20]. Furthermore, in endometrial stromal sarcoma and undifferentiated uterine sarcoma, expression of fibrillar collagens and YAP activation are associated with tumour aggressiveness [21, 22]. Simultaneously, by imposing physical constrictions, dense collagen matrices have an inhibitory effect against tumour growth and invasion [23]. However, cancer cells often acquire high collagen degradation and mechanical remodelling capabilities by which they are able to colonise neighbouring tissues and form distant metastases [10]. Matrix metalloproteinases (MMPs) are proteins with collagen degradation and remodelling functions that are central in the progression of some STS types [24].

Despite their central role in diverse tumour types including LM and other uterine sarcomas, the impact of fibrillar collagens and their downstream signalling on uLMS behaviour remains elusive. In this study, we perform an analysis of the content and morphology of fibrillar collagens in MM, LM, uLMS and other uterine sarcomas including adenosarcoma, endometrial stromal sarcoma and undifferentiated uterine sarcoma. We find that uLMS tumours present a characteristically low fibrillar collagen density with an increased number of fibre endpoints. At the gene level, we show that fibrillar collagen‐related gene expression is not lower in uLMS; instead, we observe an increase in the expression of MMPs, particularly MMP14. Using collagen‐based *in vitro* 3D cultures, we show that MMP14 activity supports uLMS proliferation. In addition, we demonstrate that uLMS cell proliferation and migration are less dependent on substrate stiffness than in MM and LM. This reduced sensitivity to stiffness may be facilitated by the observed elevated basal YAP activity at low stiffness. This study suggests that MMP14 and YAP regulate uLMS proliferation revealing them as potential biomarkers and therapeutic targets for this disease.

## 2 Materials and methods

### 2.1 Patient tissue collection (ethical permit)

The cohort used in this study contains biobanked and fresh tumour and adjacent normal tissue material from female patients who have been submitted to surgery at the Karolinska Institute Hospital, Sweden, between the years 2002 and 2021 (Stockholm medical biobank, BBK‐01615). The protocol of this study was approved by the Ethics Review Board of the Stockholm Region (DNR 2015‐143‐1), and all methods were performed in accordance with relevant guidelines and regulations and in compliance with the principles outlined in the Declaration of Helsinki. All cases were reviewed centrally. Patients gave their written informed consent.

### 2.2 Picrosirius red staining

Tissues were deparaffinised and rehydrated through graded ethanol series. Slides were incubated in picrosirius red (PSR) staining solution 1% (w/v) Direct Red 80 (2610‐10‐8; Sigma‐Aldrich, St. Louis, MO, USA) in saturated picric acid (P6744‐1GA; Sigma‐Aldrich) for 1 h at RT. Slides were washed twice with 0.5% acetic acid, dehydrated in graded ethanol series, cleared in Tissue Clear and mounted with Pertex (Histolab, Cat # 00811, Askim, Sweden). Slides were imaged with the Zeiss LSM800‐Airy confocal microscope using the 561 nm laser. Image quantification was performed with the macro TWOMBLI of fiji‐imagej [25] and the ridge detection plugin.

### 2.3 RNA extraction

Fresh‐frozen tissue samples were collected for RNA extraction. Tissue scrolls were sectioned from OCT‐embedded fresh‐frozen samples into Buffer RLT with β‐mercaptoethanol (Qiagen Sciences LLC, Germantown, MD, USA). RNA was purified following the protocol from RNeasy Micro Kit (Qiagen). RNA concentrations were determined using Qubit RNA HS kit (Thermo Fisher, Waltham, MA, USA). RNA integrity numbers (RIN) were calculated using the RNA 6000 Pico Kit 2100 Bioanalyzer (Agilent, Santa Clara, CA, USA).

### 2.4 Bioinformatic analysis

Bioinformatic analysis was conducted as previously described [26]. Specifically, preprocessing, mapping and counting RNA‐Seq reads: each fastq file was assessed using fastqc (v0.11.9) followed by adapter removing and trimming bad quality base calling with trim‐galore (v0.6.6) when it was detected in the quality report. The GRCh38.p13 human genome reference and genome annotation were downloaded (https://ftp.ncbi.nlm.nih.gov/refseq/H_sapiens/annotation/annotation_releases/109.20210226/GCF_000001405.39_GRCh38.p13/) [On May 1st, 2021]. Genome reference was indexed with hisat2 (v2.1.0) using Hierarchical Graph FM index (HGFM). At this step, only mRNA with ‘protein‐coding’ biotype in a complete genomic molecule (RefSeq NC_format), and transcripts tagged as BestRefSeq were considered. Then, reads were aligned with hisat2 using the parameter rna‐strandness of each sample after inferring the experiment using the script infer_experiment.py from rseqc (v4.0.0). Samtools was used to filter out reads unmapped, supplementary alignments and reads failing in platform/vendor quality checks and reads with mapping quality below 30 (*F* = 2828 and *q* = 30). Counting reads for each gene was performed using htseq‐count (0.13.5) with union mode and the strand‐specific information corresponding to each sample.

#### 2.4.1 Data filtering and normalisation

Genes were filtered out if less than 50% of each group (MM, LM, uLMS and other sarcomas) had count reads below 5. Next, we defined constitutive genes such as genes present in the MM group. Then, genes neither present in constitutive genes nor present in uLMS or LM groups were removed. deseq2 (r package) was used to perform a differential analysis and to generate ratios between desired comparisons. Finally, in order to present the data as a heatmap, expression values were calculated as Transcripts Per Million (TPM) using scater (r package).

### 2.5 Immunohistochemistry

The method for immunohistochemistry (IHC) staining of MMP14 was previously described [21]. Briefly, TMA sections were deparaffinised and rehydrated. Antigen retrieval was performed using 10 mmol·L^−1^ sodium citrate pH 6. Endogenous peroxidase was quenched with 0.6% H_2_O_2_ for 10 min, 2 × 5 min PBS (for ImmPRESS kit, Vector Laboratories, Newark, CA, USA), or with 0.03% H_2_O_2_ for 10 min, 1 min H_2_O and 10 min PBS [for Tyramide Signal Amplification (TSA) kit]. The ImmPRESS method was used for MMP14 staining with anti‐MT1‐MMP (LEM) antibody (MAB3328, Millipore, Burlington, MA, USA, 1 : 100). Staining of YAP (ab56701, Abcam, Cambridge, UK, 1 : 1000) was performed on tissue sections retrieved at the accredited clinical laboratory of the Department of Pathology, Karolinska University Hospital, Sweden. Staining was performed in the routine pathology laboratory using an automated Ventana Benchmark Ultra system (Ventana Medical Systems, Tucson, AZ, USA).

### 2.6 Cell culture and isolation

Human uterine leiomyosarcoma‐derived SKUT1 cells (RRID:CVCL_0533) was obtained from ATCC and validated by short tandem repeat (STR) profiling by the Karolinska Institutet in‐house service. SKUT1 cells were cultured in Eagle's Minimum Essential Medium (EMEM, Thermofisher) supplemented with 10% fetal bovine serum (FBS, Sigma), penicillin/streptomycin (Sigma) and 1× glutaMAX (Gibco, Waltham, MA, USA) at 37 °C and 5% CO_2_. Human penile primary smooth muscle cells and patient‐derived primary cells isolated from normal myometrium, uterine leiomyoma and uterine leiomyosarcoma tumours were cultured in primary Smooth Muscle Cell Basal Medium (Sigma) and penicillin/streptomycin, 10% FBS, 0.5 ng·mL^−1^ EGF (Sigma), 2 ng·mL^−1^ bFGF (Sigma) and 5 μg·mL^−1^ insulin (Sigma) with at 37 °C and 5% CO_2_. Cells were tested for mycoplasma routinely.

Isolation of patient‐derived cells was performed from fresh tissue samples selected by an experienced pathologist. Tissues were cut into 1–2‐mm pieces and subsequently incubated in serum‐free medium containing collagenase/hyaluronidase (Stem Cell Technologies, Vancouver, Canada) at 37 °C. After tissue digestion, the solutions were washed with DPBS (Gibco), and red blood cells were lysed with Tris‐buffered ammonium chloride solution. After 2× washes with PBS and 2× washes with complete culture medium, cells were seeded on plates coated with 50 μm·mL^−1^ rat tail collagen I (Sigma).

### 2.7 Collagen‐I polyacrylamide hydrogels

Round microscopy cover slides were washed with 70% ethanol and 0.1 m NaOH, covered with 3‐aminopropyltrimethoxysilane (3‐APTS, Sigma) for 3 min for activation, incubated for 30 min in 0.5% glutaraldehyde (Sigma), and washed in sterile MilliQ water. Polyacrylamide solutions containing acrylamide monomers (Sigma), crosslinker *N*,*N*‐methylene‐bis‐acrylamide (Sigma) and PBS in different concentrations were prepared to create different Young's modulus (0.5, 2, 4.5, 10, 20 and 115 kPa) as previously described [27]. 5 μL of 10% ammonium persulfate (Sigma) and 0.75 μL *N*,*N*,*N*′,*N*′‐tetramethylethylenediamine (Sigma) were added into 0.5 mL mixtures, and one drop of the mixture was placed on rain repellent‐treated microscopy slides, and the activated cover slides were placed on top. After polymerisation (3–10 min), the cover slides with polyacrylamide gels were washed with PBS. Next, the cover slides were treated with 1 mg·mL^−1^ *N*‐sulfosuccinimidyl‐6‐(4′‐azido‐2′‐nitrophenylamino) hexanoate (Sigma) and exposed to ultraviolet (UV) light to allow subsequent collagen‐I binding. The cover slides were incubated at room temperature with 10 μg·mL^−1^ rat‐tail collagen‐I (Sigma) for 3 h, washed with PBS and placed in UV light for sterilisation. Wells containing cover slides were seeded with SKUT1 cells or primary SMC, uLMS, LM or MM cells.

### 2.8 Tissue analysis of mitoses

Per each case, mitotic figures were counted in 10 consecutive high‐power fields (400× magnification). The sum of mitotic figures defined the “mitotic count” for each case.

### 2.9 Immunofluorescence and confocal imaging

Cells were fixed directly on the hydrogel‐cover slide in 4% paraformaldehyde for 20 min at RT, permeabilized with ice‐cold acetone:methanol (1 : 1) for 45 s for nuclear staining and blocked in 5% bovine serum albumin (BSA, Biowest, Nuaillé, France) for membrane staining or 5% BSA‐0.3% Triton‐X (Sigma) for nuclear staining, for 30 min at RT. The cells were incubated with primary antibodies diluted in blocking buffer for 2 h at RT. After washing, the cover slides were incubated in secondary antibodies and phalloidin for 1 h. EdU staining (Click‐iT Plus EdU Imaging Kit, Thermo‐Fisher) was performed as described by the manufacturer. Cover slides were washed and mounted on microscopy slides with VECTASHIELD Antifade Mounting Medium with DAPI (Vector Laboratories, Newark, CA, USA). Images were taken with a Zeiss LSM 800 confocal microscope with Airyscan and were analysed in imagej (fiji).

### 2.10 Inhibitor treatments and cell viability assay

Cells were treated with GM6001 (pan‐MMP; Calbiochem, San Diego, CA, USA), NSC405020 (MMP14‐specific; Selleckchem, Houston, TX, USA) or verteporfin (YAP; Sigma) for the indicated times and concentrations. Cytotoxicity was assessed after 48 h using CellTiter‐Glo 3D Luminescent Cell Viability Assay (Promega, Madison, WI, USA) for 30 min before luminescence detection.

### 2.11 siRNA knockdowns

For knockdown, siRNAs against human MMP14(1) (L‐004145‐00‐0005; Dharmacon, Lafayette, CO, USA), MMP14(2) (SI00071176, Qiagen), YAP1(1) (SI02662954 and SI04438637; GeneSolution, Qiagen), YAP1(2) (SI04438644 and SI04438651; GeneSolution, Qiagen) or nontargeting control siRNA (SI03650318; Qiagen) were used. Briefly, 200 μL optiMEM and 0.75 or 1.5 μL Lipofectamine 3000 (Thermofisher) were mixed and incubated for 5 min and a mixture of 200 μL optiMEM with 20 pmol siRNA were added and incubated for 15 min. Then, the mixtures were added to 1 mL serum and antibiotic‐free medium. After 48 h in culture cells were used for the following experiments.

### 2.12 Antibodies

For immunofluorescence, primary antibodies used were Ki67 (8D5; #9449; Cell Signaling Technology, Danvers, MA, USA; 1 : 800), active integrin b1 (12G10; ab30394; Abcam; 1 : 400), YAP/TAZ (63.7; sc‐101199; Santa Cruz Biotechnology, Dallas, TX, USA; 1 : 200), cleaved‐caspase 3 (Asp175; #9664 Cell Signalling Technology; 1 : 800). Secondary andibodies for immunofluorescence were goat anti‐rabbit Alexa Fluor Plus 555 (#A32732, ThermoFisher Scientific; 1 : 1000) and goat anti‐mouse Alexa Fluor Plus 499 (#A32731, ThermoFisher Scientific; 1 : 1000).

### 2.13 Live migration analysis

All live cell imaging was conducted in a Cytation 5 imaging reader at +37 °C and 5% CO_2_ (BioTek™ CYT5MPV, Santa Clara, CA, USA). Images were taken at 2‐h time intervals over 3 days. For analysis, the manual tracking plugin in imagej (fiji) was used. The distinct cell migration parameters were analysed with the software diper [28].

## 3 Results

### 3.1 uLMS tumours are characterised by low fibrillar collagen density and high‐fibre endpoints

**Fig. 1:**
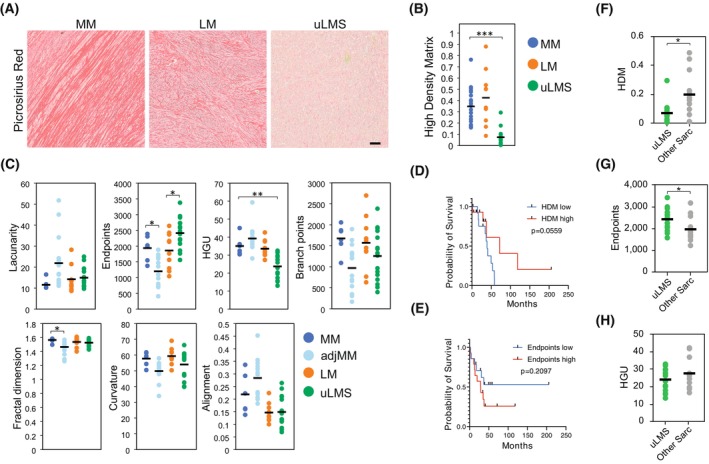
uLMS are characterised by low fibrillar collagen density and high fibre endpoints. (A) Representative images of picrosirius red staining of normal myometrium (MM; *n* = 6), uterine leiomyoma (LM; *n* = 10) and uterine leiomyosarcoma (uLMS; *n* = 16) tissues. Scale bar indicates 100 μm. (B) Quantification of the fraction of high‐density matrix (HDM) in tissues from (A). One‐way ANOVA with *post hoc* Tukey's test. (C) Quantification of morphological aspects of the collagenous matrix based on staining from (A) and including tumour‐adjacent MM (adjMM, < 1 mm from tumour edge; *n* = 14). HGU (hyphal growth unit). One‐way ANOVA with *post hoc* Tukey's test. (D, E) Kaplan–Meier curves of uterine sarcoma patients grouped based on their fraction of HDM (D) or number of fibre endpoints (E) (low: *n* = 14; high: *n* = 14). Log‐rank test. (F–H), Quantification of HDM (F), fibre endpoints (G), and HGU (H) comparing uLMS tumours (*n* = 16) and the other sarcomas included in our cohort (other Sarc; *n* = 12). Two‐tailed Student's *t*‐test. Each dot in the graphs indicates the average of four measurements per tumour sample; horizontal bar indicates the average of the tissue type. *P < 0.05, **P < 0.01, ***P < 0.001.

To understand the function of fibrillar collagens on uterine leiomyosarcoma (uLMS), we explored the differences in fibrillar collagen density and network morphology between normal myometrium (MM), uterine leiomyoma (LM), and uLMS tissues. Analysis of picrosirius red‐stained tissues with the fiji macro TWOMBLI [25] revealed that uLMS tissues present a lower fraction of high‐density matrix (HDM) regions, indicating that uLMS tissues have lower collagen density (Fig. 1A,B). Moreover, uLMS tissues presented increased fibre endpoints and reduced hyphal growth unit (HGU), a measure of the number of endpoints per unit length (Fig. 1C), showing that collagen fibres in uLMS are shorter and more fragmented. Comparing the MM at distant or tumour‐adjacent (0–1 mm from the tumour edge) locations showed reduced fractal dimension and endpoints in tumour‐adjacent MM, and a nonsignificant trend to increased fibre alignment and lacunarity and decreased curvature and branchpoints (Fig. 1C and Fig. S1). These alterations in the density and morphology of tumour‐adjacent collagen may result from tensile solid stresses originated by the constant increase in tumour volume [23].

To investigate whether the observed differences in fibrillar collagens found in uLMS are associated with tumour aggressiveness, we compared the overall survival of a cohort of uterine sarcoma patients (*n* = 28; Table S1) presenting distinct HDM and fibre endpoints. Uterine sarcomas with lower HDM and higher endpoints showed a nonsignificant trend to reduced overall survival (HDM *P* = 0.0559, endpoints *P* = 0.2097; Fig. 1D,E). Concurrently, the frequency of metastasis was higher in uLMS patients with low HDM primary tumours (87.5% in low HDM versus 37.5% in high HDM) and in patients with high endpoints (50% in low versus 75% in high endpoints; Fig. S2). To further investigate whether the fibrillar collagen characteristics are a differential trait of uLMS, we compared uLMS tissues with the other uterine sarcomas included in our cohort (adenosarcoma, endometrial stromal sarcoma, undifferentiated uterine sarcoma and PEComa). In uLMS, HDM was lower and the number of endpoints was higher than in other sarcomas, but no significant differences were observed in HGU (Fig. 1F–H). These results indicate that uLMS tumours present a particularly low density of fibrillar collagens, contain more fragmented fibres and suggest a link between reduced collagen density and uLMS tumour aggressiveness.

### 3.2 MMP14 activity supports uLMS cell proliferation in collagenous microenvironments

**Fig. 2:**
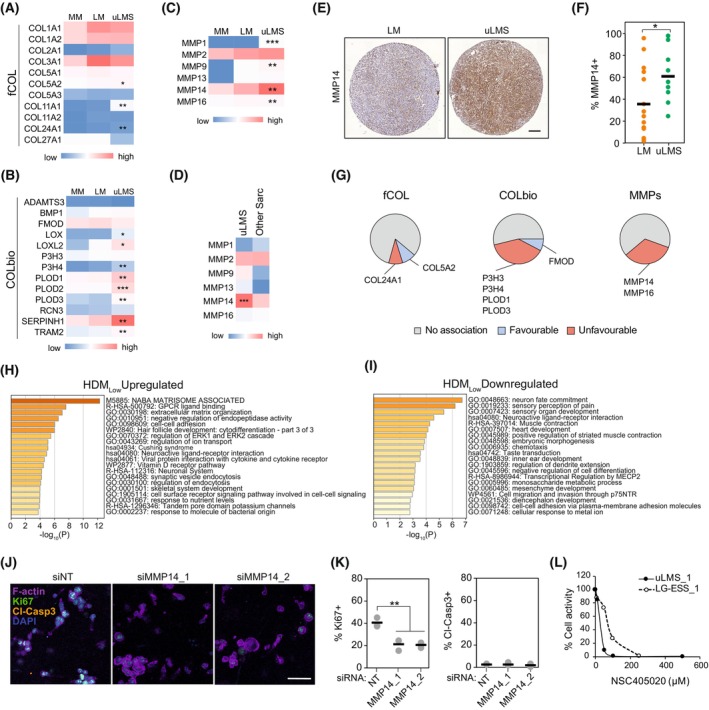
uLMS cells highly express MMP14 and depend on its activity for proliferation in collagenous environments. (A–C), Relative average expression of fibrillar collagen (fCOL) (A), collagen biosynthesis and crosslinking (COLbio) (B), and collagen‐cleaving matrix metalloproteinases (MMPs) (C), in normal myometrium (MM; *n* = 31), uterine leiomyoma (LM; *n* = 28), and uterine leiomyosarcoma (uLMS; *n* = 17). One‐way ANOVA with *post hoc* Tukey's test. (D) Relative average expression of MMPs in uLMS and other uterine sarcomas included in the cohort (other Sarc; *n* = 17). Two‐tailed Student's *t*‐test. (E) Representative examples of MMP14 protein staining in LM (*n* = 12) and uLMS (*n* = 9) tissues. Scale bar indicates 100 μm. (F) Quantification of the percentage of the area of the tissue positive for MMP14 in LM (*n* = 12) and uLMS (*n* = 9) tissues, indicating higher MMP14 protein expression in uLMS. Each datapoint represents one patient; horizontal bars indicate the average per tissue type. Two‐tailed Student's *t*‐test. (G) Association between high expression of fCOL, COLbio and MMP genes and uLMS patient prognosis of the TCGA cohort (*n* = 24). Patients groups were generated by the best cut‐off method, favourable/unfavourable survival was defined as significant changes of median survival (*P* < 0.05). (H, I) Metascape pathway analysis of the genes upregulated (H) and downregulated (I) in uLMS tumours with low fraction of high‐density matrix (HDMl^ow^; *n* = 10) compared with HDM^high^ (*n* = 8) uLMS tumours. (J) Representative images of SKUT1 cells in 3D collagen gels 4 days after MMP14 knockdown (*n* = 3 independent experiments). Scale bar indicates 50 μm. (K) Proliferative (Ki67+) and apoptotic (Cleaved‐caspase 3+) cell quantification from (J) (*n* = 3 independent experiments). One‐way ANOVA with *post hoc* Tukey's test. (L) Quantification of the response of patient‐derived uLMS and low‐grade endometrial stromal sarcoma (LG‐ESS) cells to NSC405020. Each data point indicated the average of 3 technical replicates. *P < 0.05, **P < 0.01, ***P < 0.001.

To explore the origin of the reduced collagen density found in uLMS tumours, we compared the expression of fibrillar collagens and collagen biosynthesis and crosslinking‐related genes in MM, LM and uLMS tissues. The expression of three of 11 fibrillar collagen and eight of 13 collagen biosynthesis genes was upregulated in uLMS compared with MM and LM (Fig. 2A,B), suggesting that the low density of fibrillar collagens observed in uLMS is not caused by a reduced expression of fibrillar collagen‐related genes. Next, we investigated whether uLMS tumours present differential expression of collagen‐degrading MMPs. Compared with MM and LM, uLMS tumours showed increased expression of four of six genes encoding collagen‐degrading MMPs, including *MMP14* as the most highly expressed MMP in uLMS (Fig. 2C). Similarly, compared with other uterine sarcomas, *MMP14* expression was upregulated in uLMS (Fig. 2D). These results were confirmed at the protein level *in situ*, showing higher MMP14 expression in uLMS than in benign LM tumours (Fig. 2E,F).

Next, we evaluated the prognostic value of fibrillar collagens, collagen biosynthesis‐related and MMP gene expression in uLMS. High expression of two of 11 fibrillar collagen genes was associated with patient overall survival (*COL24A1* unfavourable and *COL5A2* favourable association; based on median survival, best cut‐off patient grouping with *P* < 0.05), whilst four of 13 collagen biosynthesis/crosslinking genes were associated with unfavourable prognosis (*P3H3*, *P3H4*, *PLOD1*, and *PLOD3*) and one of 13 with favourable prognosis (*FMOD*; Fig. 2G). The expression of *MMP14* and *MMP16* was also associated with unfavourable prognosis in uLMS patients. Thus, the expression of various collagen‐remodelling and degrading genes is linked to uLMS aggressiveness. To further understand the origin of reduced fibrillar collagen density in uLMS, we compared the gene expression of uLMS tumours with high and low HDM. Pathway analysis of genes upregulated in low HDM tumours indicated that the ‘NABA Matrisome Associated’ was the most significantly altered gene set followed by ‘GPCR ligand binding’, and ‘extracellular matrix organisation’ (Fig. 2H). Amongst the upregulated matrisome‐associated genes in low HDM uLMS tumours, we found the ECM‐degrading MMP13 and ADAMTS20 (Table S2), further suggesting increased ECM proteolysis in uLMS tumours. Contrarily, pathway analysis of genes downregulated in low HDM uLMS tumours did not reveal any ECM‐related pathway (Fig. 2I). These results indicate that the low collagen density in uLMS tumours is coupled with an enhanced collagen remodelling and degradation.

Finally, to evaluate the function of MMPs and, in particular, MMP14 in uLMS, we embedded uLMS cells in 3D collagen I‐based matrices, mimicking collagen‐rich environments such as the MM (Fig. S3A). In the uLMS transformed cell line SKUT1, MMP14 knockdown decreased cell proliferation, indicated by Ki67 positivity, without affecting cell apoptosis, shown by cleaved‐caspase 3 expression (Fig. 2J,K and Fig. S3B). Similarly, MMP inhibition with the broad‐spectrum inhibitor GM6001 affected cell distribution, inducing the formation of cell clusters compared with the single‐cell invasive growth pattern observed in the control, and reduced total cell activity, indicative of reduced proliferation (Fig. S3C). Specifically, inhibiting MMP14 with NSC405020 caused a more pronounced reduction in SKUT1 cell growth, as observed by the lower cell density and reduced total viability, compared with GM6001. Next, we evaluated the response of patient‐derived uLMS cells to MMP14 inhibition and compared it with the response of less aggressive low‐grade endometrial stromal sarcoma (LG‐ESS) cells. Inhibition of MMP14 in uLMS caused a drastic reduction in cell spreading and growth, indicated as reduced relative cell activity, an effect that was less pronounced in the LG‐ESS cells (Fig. S3D and Fig. 2L). Altogether, these results indicate that, as in various carcinoma and in fibrosarcoma cells, MMP14 activity is essential for the proliferation and aggressive growth pattern of uLMS cells in collagen‐rich environments [29].

### 3.3 Proliferation of uLMS cells is characterised by reduced substrate stiffness dependence

**Fig. 3:**
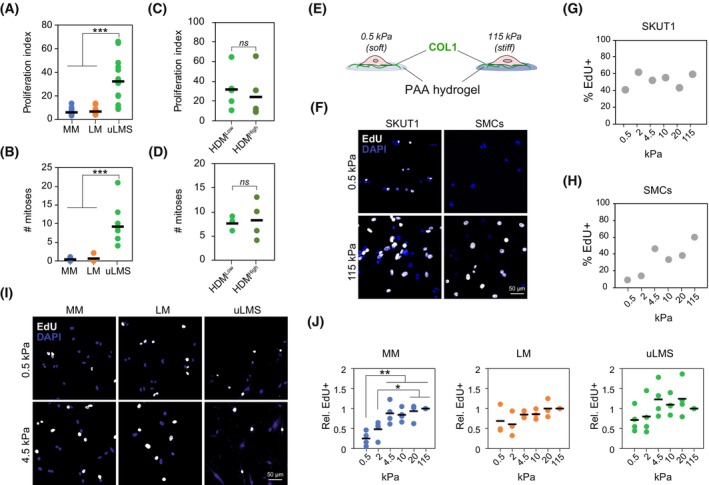
Adhesion to collagen substrates has minimal effect on uLMS cell proliferation. (A) Gene expression‐based proliferation index in normal myometrium (MM; *n* = 31), leiomyoma (LM; *n* = 28) and uterine leiomyosarcoma (uLMS; *n* = 20) tissues indicating enhanced proliferation in uLMS. One‐way ANOVA with *post hoc* Tukey's test. (B) Number of mitoses per field in MM (*n* = 7), LM (*n* = 6) and uLMS (*n* = 10) tissues. One‐way ANOVA with *post hoc* Tukey's test. (C, D) Proliferation index (C) and number of mitoses per field (D) in uLMS tumours with high (*n* = 4) and low (*n* = 5) high‐density matrix (HDM) indicating no significant differences between groups. Each dot represents one patient tissue; horizontal bars indicate average per group. Two‐tailed Student's *t*‐test. (E) Schematic representation of the 2D model of controlled collagen substrate stiffness based on polyacrylamide (PAA) hydrogels. (F–H) Representative images (scale bar indicates 50 μm) and quantification of cell proliferation measured by EdU incorporation in SKUT1 cells (*n* > 95 cells per condition) (G) and primary smooth muscle cells (SMCs; *n* > 60 cells per condition) (H) at indicated PAA hydrogel stiffness. (I, J) Representative images (I) and quantification (J) of EdU incorporation in MM cells (*n* = 4 donors), LM (*n* = 3 donors) and uLMS (*n* = 4 donors) cells adhered to PAA hydrogel substrates with indicated stiffness. Scale bar indicates 50 μm. Each dot indicates the average per donor; horizontal bars indicate the average per tissue type. One‐way ANOVA with *post hoc* Tukey's test. *ns P* > 0.05, *P < 0.05, **P < 0.01, ***P < 0.001.

Cell adhesion to a stiff collagenous ECM impacts cell function, including changes in cell proliferation, phenotype, survival and motility through integrin signalling and other mechanically activated pathways [30]. As uLMS tumours present low fibrillar collagen density, we hypothesised that cell functions in uLMS are less sensitive to stiff collagen‐inducing signalling than in normal MM and benign LM cells. To investigate the effect of fibrillar collagens on uLMS cell proliferation, we first compared the cell proliferation in uLMS tumours with normal MM and benign LM. As expected, uLMS tumours showed enhanced proliferation index, calculated as the combined expression of multiple proliferation‐related genes [31], and higher mitotic number than MM and LM tissues (Fig. 3A,B). Comparing the proliferation of uLMS tumours with high and low HDM showed no differences in proliferation index or mitotic number (Fig. 3C,D), indicating that the collagen density of uLMS tumours does not correlate with cell proliferation *in vivo*. To functionally evaluate the effect of substrate adhesion and stiffness on uLMS cell proliferation, we seeded SKUT1 cells or normal human smooth muscle cells (SMCs) on collagen I‐functionalized polyacrylamide hydrogels of increasing stiffness, from very soft 0.5 kPa to supraphysiologically stiff 115 kPa (Fig. 3E). The proliferation of SKUT1 cells, indicated by EdU incorporation, showed little variations in the different substrate stiffness (Fig. 3F,G). However, SMCs showed a progressive increase in EdU+ cells with increasing substrate stiffness (Fig. 3F,H). Similarly, while EdU incorporation steadily increased with substrate stiffness in patient‐derived MM cells, this effect was less pronounced in LM and uLMS cells (Fig. 3I,J). In general, the highest proliferation was achieved at physiological stiffness (> 4.5 kPa). However, uLMS cell proliferation showed high variability between donors, suggesting that the proliferation of some uLMS cells is more substrate stiffness‐dependent than others. Altogether, these results show that the proliferation of uLMS cells presents reduced dependency on collagen adhesion and substrate stiffness.

### 3.4 Proliferation is supported by enhanced YAP activity in uLMS cells

**Fig. 4:**
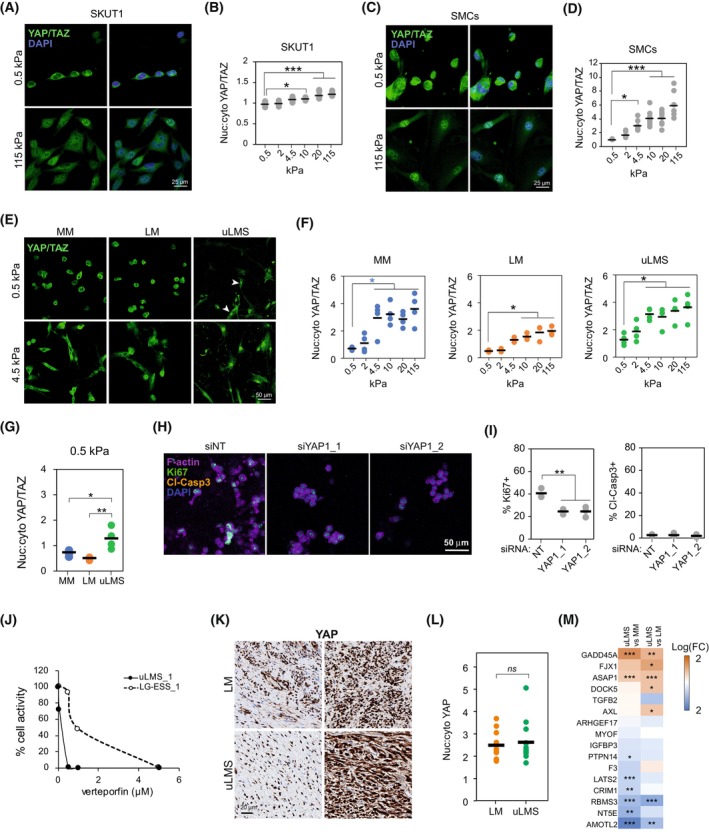
Enhanced YAP activation in uLMS supports cell proliferation in soft collagenous substrates. (A, B) Representative images (A) and quantification (B) of YAP/TAZ subcellular localisation in SKUT1 cells adhered to PAA hydrogels at indicated stiffness (*n* > 71 cells per condition). One‐way ANOVA with *post hoc* Tukey's test. Scale bar indicates 25 μm. (C, D) Representative images (C) and quantification of YAP/TAZ subcellular localisation in primary smooth muscle cells (SMCs) adhered to PAA hydrogels at indicated stiffness (*n* > 49 cells per condition). One‐way ANOVA with *post hoc* Tukey's test. Scale bar indicates 25 μm. (E, F) Representative images (E) and quantification (F) of YAP/TAZ subcellular localisation in normal myometrium (MM; *n* = 4 donors), leiomyoma (LM; *n* = 3 donors), and uterine leiomyosarcoma (uLMS; *n* = 4 donors) cells adhered to indicated stiffness. One‐way ANOVA with *post hoc* Tukey's test. Arrowheads indicate cells with nuclear YAP/TAZ. Scale bar indicates 50 μm. (G) Comparison of YAP/TAZ nuclear:cytoplasmic ratio in MM (*n* = 4 donors), LM (*n* = 3 donors), uLMS (*n* = 4 donors) cells adhered to soft 0.5 kPa PAA hydrogels showing increased nuclear YAP/TAZ in uLMS cells. One‐way ANOVA with *post hoc* Tukey's test. (H) Representative examples of SKUT1 cells embedded in 3D collagen matrices 4 days after YAP siRNA knockdown (*n* = 3 independent experiments with more than 500 cells counted per condition). Scale bar indicates 50 μm. (I) Proliferative (Ki67+) and apoptotic (Cleaved‐caspase 3+) cell quantification from (H) (*n* = 3 independent experiments with more than 500 cells counted per condition). One‐way ANOVA with *post hoc* Tukey's test. (J) Quantification of the response of 3D collagen‐embedded patient‐derived uLMS and low‐grade endometrial stromal sarcoma (LG‐ESS) to verteporfin based on total ATP content (*n* = 3 technical replicates). (K, L) Representative images showing two distinct examples of LM and uLMS tissues stained for YAP (K) and quantification of nuclear:cytoplasmic YAP ratio showing no difference between the tissues (LM *n* = 10 and uLMS *n* = 14). Scale bar indicates 20 μm. Two‐tailed Student's *t*‐test. (M) Differential gene expression of YAP target genes between uLMS and MM and between uLMS and LM tissues. Two‐tailed Student's *t*‐test. *P < 0.05, **P < 0.01, ***P < 0.001.

To further evaluate the lower dependence of uLMS cells to collagen adhesion and substrate stiffness, we investigated the subcellular localisation of the mechanotransducers and proliferation regulators YAP/TAZ, found in the nucleus in their active form, which is associated with undifferentiated uterine sarcoma aggressiveness [22]. Nuclear localisation of YAP/TAZ in SKUT1 increased with stiffness (Fig. 4A,B). Similarly, YAP/TAZ nuclear localisation was higher with increasing stiffness in SMCs, although their response to increasing stiffness was more pronounced than in SKUT1 (Fig. 4C,D). In MM, LM and uLMS patient‐derived cells, nuclear YAP/TAZ progressively increased with higher stiffness (Fig. 4E,F). However, unlike in MM and LM cells, the average YAP/TAZ nuclear:cytoplasmic ratio in uLMS at 0.5 kPa was > 1, indicating mostly nuclear localisation (Fig. 4E,G). To investigate whether YAP activity is necessary for uLMS cell proliferation, we treated SKUT1 cells with the YAP inhibitor verteporfin. Verteporfin treatment reduced the spreading of SKUT1 cells within 3D collagen matrices and the percentage of proliferating, EdU+, cells (Fig. S4A,B). Furthermore, the percentage of apoptotic cells, indicated by cleaved‐caspase 3 positivity, was reduced by verteporfin, demonstrating that the lower proliferation was not the result of enhanced apoptosis (Fig. S4C). Similarly, YAP siRNA knockdown (Fig. S4D) reduced the percentage of Ki67+ cells without affecting the percentage of apoptotic, cleaved‐caspase 3+ cells (Fig. 4H,I). Likewise, verteporfin had a strong negative effect on patient‐derived uLMS cell growth, whilst this effect was less pronounced in the LG‐ESS cells used for comparison (Fig. 4J). These results indicate that YAP activity regulates uLMS cell proliferation. Finally, to evaluate whether YAP is active in uLMS tumours despite the low collagen density, we analysed the YAP subcellular localisation in LM and uLMS tumours. Subcellular localisation of YAP was mostly nuclear in both LM and uLMS tumours (Fig. 4K,L). Moreover, the expression of YAP target genes in uLMS compared with MM and LM tissues did not reveal specific trends, although various differentially expressed genes were observed (Fig. 4M). Altogether, these results indicate that the uLMS proliferation‐supporting YAP activity is less dependent on substrate stiffness than in MM and LM cells. This enhanced basal YAP activity may facilitate uLMS growth in soft, low collagen microenvironments.

### 3.5 uLMS cells present increased integrin and YAP‐activating gene expression

**Fig. 5:**
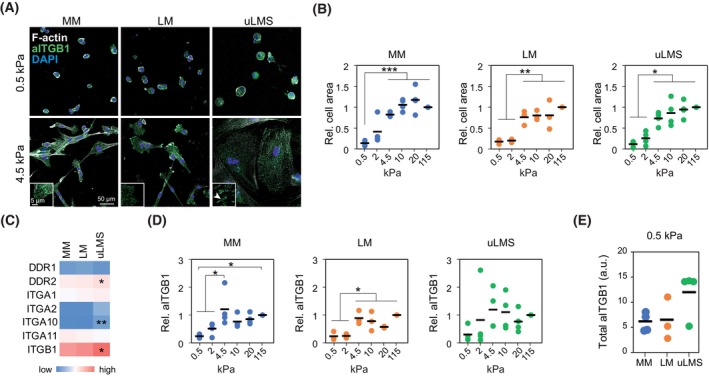
uLMS cells overexpress collagen receptors and show enhanced integrin β1 activity. (A) Representative images of normal myometrium (MM), leiomyoma (LM) and uterine leiomyosarcoma (uLMS) cells adhered to PAA hydrogels showing distinct morphology and presence of focal adhesions at indicated stiffness. At least 50 cells were counted per condition and patient. Scale bar indicates 50 μm. Insets show higher magnification of example cellular edges with elongated focal adhesion in uLMS (arrowhead). Inset Scale bar indicates 5 μm. (B) Quantification of spreading cell area of MM (*n* = 4 donors), LM (*n* = 3 donors), uLMS (*n* = 4 donors) cells on the indicated substrate stiffness. At least 50 cells were counted per condition and patient. (C) Relative collagen receptor gene expression in MM (*n* = 31), LM (*n* = 28) and uLMS (*n* = 17) tissues showing enhanced expression in uLMS of indicated genes. (D) Change in active integrin β1 intensity (aITGB1) at distinct polyacrylamide (PAA) hydrogel stiffness relative to 115 kPa in MM (*n* = 4 donors), LM (*n* = 3 donors), uLMS (*n* = 4 donors) cells. (E) Comparison of total aITGB1 intensity in MM (*n* = 4 donors), LM (*n* = 3 donors), and uLMS (*n* = 4 donors) cells at 0.5 kPa showing a nonsignificant increase in uLMS cells. All statistical analyses were performed by one‐way ANOVA with *post hoc* Tukey's test. *P < 0.05, **P < 0.01, ***P < 0.001.

To investigate whether the reduced uLMS dependence on collagen adhesion derives from direct alterations in collagen adhesion and substrate stiffness sensing, we compared the relative cell spreading and adhesion of MM, LM and uLMS cells at distinct stiffness. In all cell types, cell spreading area increased with stiffness, with a significant increase between 0.5 and 4.5 kPa, which approximates the stiffness of normal MM tissue (Fig. 5A,B). However, at 4.5 kPa, visible and elongated focal adhesions were only observed in uLMS cells (Fig. 5A), indicating a lower stiffness threshold for the formation of these signalling hubs. To understand this difference, we compared the gene expression of collagen I receptors between tissue types. Expression of *DDR2*, *ITGA10* and *ITGB1* was higher in uLMS than in MM and LM (Fig. 5C). Next, we investigated whether the collagen substrate stiffness‐induced activation of integrin β1 was different in MM, LM, and uLMS cells. Expression of active integrin β1 increased in MM and LM cells with stiffness, significantly at > 4.5 kPa compared to 0.5–2 kPa (Fig. 5D). However, the response of uLMS cells from distinct tumours was heterogeneous, although a trend of increasingly active integrin β1 was also observed (Fig. 5D). Finally, we compared the levels of active integrin β1 at soft 0.5 kPa in the distinct cell types, which showed a nonsignificant trend to higher active integrin β1 in uLMS cells (*P* = 0.1813; Fig. 5E). These results indicate that there is a higher expression of collagen receptors in uLMS cells and a lower stiffness threshold for focal adhesion formation, which could be involved in the enhanced activation of YAP and reduced collagen adhesion dependence to cell proliferation. However, other YAP‐activating factors such as the enzymes of the mevalonate pathway 3‐Hydroxy‐3‐Methylglutaryl‐CoA Reductase and squalene epoxidase [32] also appeared upregulated in uLMS at the gene level (Fig. S5), suggesting that multiple factors contribute to the enhanced YAP activation in uLMS.

### 3.6 uLMS cell migration shows reduced response to collagen stiffness

**Fig. 6:**
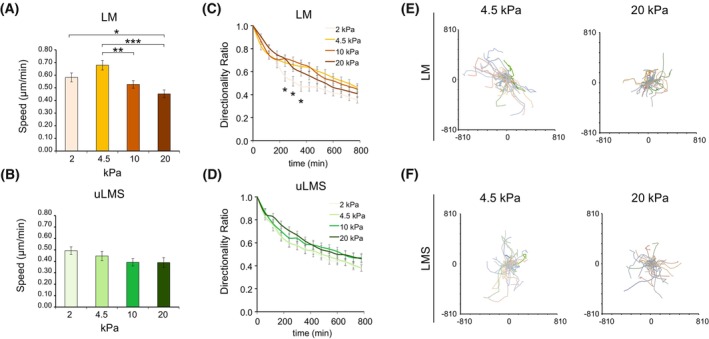
uLMS cell migration is not affected by collagen substrate stiffness. (A, B) Cell migration speed of leiomyoma (LM) cells (A) and uterine leiomyosarcoma (uLMS) cells (B) at indicated polyacrylamide (PAA) hydrogel stiffness (*n* > 10 cells/stiffness per donor; LM *n* = 3 donors and uLMS *n* = 4). (C, D) Directionality ratio of LM (C) and uLMS (D) cells at distinct PAA hydrogel stiffness (*n* > 10 cells/stiffness per donor; LM *n* = 3 donors and uLMS *n* = 4). (E, F) Individual cell trajectories of LM (E) and uLMS (F) cells at indicated stiffness showing relatively shorter trajectories of LM cells at 20 kPa (*n* > 10 cells/stiffness per donor; LM *n* = 3 donors and uLMS *n* = 4). Bar graphs represent average ± SD. All statistical analyses were performed by one‐way ANOVA with *post hoc* Tukey's test. *P < 0.05, **P < 0.01, ***P < 0.001.

Cancer cell migration is a process linked to tumour aggressiveness that is regulated by ECM adhesion and substrate stiffness [33]. To investigate whether the impact of substrate stiffness on uLMS migration, as with proliferation, is reduced, we seeded patient‐derived uLMS and LM cells on gels ranging from 2 to 20 kPa and tracked them over 13 h. Benign LM cells showed the highest migration speed at 4.5 kPa (Fig. 6A). Instead, uLMS cell migration speed did not significantly vary across the range of stiffness investigated, although a trend towards reduced speed with increasing stiffness was observed (Fig. 6B). Analysis of directionality ratio, a measure that indicates how persistent the migration of a cell is, showed lower directionality ratio of LM cells at 2 kPa (Fig. 6C). However, the directionality of uLMS cells remained unaffected by substrate stiffness (Fig. 6D). Furthermore, the paths followed by LM cells at 4.5 kPa were generally longer than at 20 kPa, whilst this difference was not observed in uLMS cells (Fig. 6E,F). These results indicate that, within the *in vivo*‐relevant stiffness range studied, LM cell migration is stiffness‐dependent while uLMS migration is stiffness‐insensitive.

## 4 Discussion

Various clinical trials are currently evaluating the therapeutic potential of targeting collagens and their downstream signalling to treat carcinomas [34]. These treatments could be an option for LM and specific uterine sarcoma subtypes, as enhanced collagen deposition and YAP activity are associated with undifferentiated uterine sarcoma and endometrial stromal sarcoma aggressiveness [21, 22]. However, due to the lack of knowledge on the relationship between the collagenous ECM and uLMS cells, the potential benefit of targeting collagen‐related molecules in uLMS has not been explored.

Here, we show that, unlike LM tumours, uLMS present low fibrillar collagen density with higher fibre endpoints. Moreover, lower collagen density correlates with a trend to reduced overall patient survival and increased metastasis. These results suggest that, unlike the aforementioned carcinomas and non‐uLMS uterine sarcomas, lower fibrillar collagen expression is associated with tumour aggressiveness. Thus, in principle, uLMS patients may not benefit from collagen‐reducing therapies. This characteristic of uLMS is uncommon but not unique. For instance, in pancreatic cancer, collagen type I deposition is associated with reduced aggressiveness as it acts as a shield against pro‐tumour immunity [35].

We find that fibrillar collagen‐related genes are not downregulated in uLMS. Furthermore, we show that uLMS tumours highly express various collagen‐remodelling genes, including collagen‐crosslinking and matrix metalloproteinase genes, particularly *MMP14*. This high expression of collagen biosynthesis and degradation genes suggests that uLMS tumours present increased collagen turnover. The enhanced aggressiveness in low collagen‐uLMS may be given by the high collagen remodelling and degradation, resulting in uLMS cell invasiveness, colonisation of neighbouring tissues and the formation of metastases [24]. Alternatively, the altered collagenous matrix can lead to increased aggressiveness through the regulation of tumour immunity and other factors of the tumour microenvironment such as the vasculature [36], which should be further explored to fully understand the function of collagen and the potential of targeting collagen‐related proteins in uLMS.

We show that MMP14 inhibition and knockdown reduces uLMS cell proliferation in collagenous matrices and that its gene expression is associated with unfavourable prognosis. The function of MMP14 in uLMS proliferation can derive from the reduction in proliferation‐inhibiting mechanical confinement as a result of collagen degradation [37]. However, other noncollagenous matrix molecules found in tumours, which are not MMP‐degradable, can also induce mechanical confinement. Instead, MMP14 activity could be mediating the activation of proliferation‐inducing factors such as the heparin‐binding EGF‐like growth factor, involved in uLMS cell survival [38, 39, 40]. Combining *ex vivo* cultures or decellularized tumours, representing the real TME in uLMS, may shed light on the function of MMP14 in tumours. Moreover, the use of MMP‐cleavable and noncleavable matrices could discern between the mechanical confinement and cell signalling function of MMP14 on uLMS cell proliferation. Nevertheless, our results indicate that MMP14 is a potential target for the treatment of uLMS. However, to date, the usefulness of specific MMP14 inhibitors in cancer treatment has not been demonstrated clinically, and the failure of previous clinical trials has been partly attributed to the inhibition of antitumour functions of MMP14 [34, 41, 42].

Enhanced collagen degradation can lead to reduced matrix stiffness and adhesion ligand availability, which are associated with reduced cell proliferation and migration [30, 32, 33]. However, here, we show that the impact of collagen adhesion on uLMS cell proliferation and migration is reduced compared with LM and MM. Interestingly, we show that functional YAP, a central regulator of cell proliferation downstream of ECM adhesion [13, 17], supports uLMS proliferation. Similarly, the YAP inhibition caused a round shape in uLMS cells, indicative of reduced invasiveness potential in cells with mesenchymal characteristics such as uLMS cells. Although the function of YAP on the reduced uLMS cell migration sensitivity to substrate stiffness was not directly investigated, previous reports show that YAP activity regulates stiffness‐dependent migration [43, 44]. Moreover, we find that YAP is overactivated in uLMS cells adhered to soft substrates compared with MM and LM. This enhanced YAP activation could derive from the enhanced expression of collagen receptors, the increased activity of other positive regulators of YAP such as the mevalonate pathway or the reduced activity of YAP‐inhibiting molecules [32, 45], although their direct involvement on YAP activity were not investigated here. Unravelling the molecular mechanisms behind the enhanced YAP activity in uLMS tumours will be crucial to determine its function and the therapeutic potential of YAP regulators. Altogether, these results suggest that inhibiting YAP, either directly or indirectly through YAP‐regulatory molecules, could be beneficial for uLMS treatment. Moreover, as YAP is involved in chemotherapy resistance, YAP inhibition combined with otherwise poorly effective chemotherapeutics, should be considered [46, 47].

## 5 Conclusions

In conclusion, the results presented here indicate that uLMS tumours present a particularly reduced fibrillar collagen content, likely derived from high collagen matrix turnover, associated with tumour aggressiveness. Furthermore, the enhanced YAP activity in uLMS tumours supports cell proliferation in their native low‐collagen microenvironment. Thus, these results highlight interesting avenues for understanding the biological role of collagen in uLMS progression and for targeting collagen remodelling and YAP signalling for the treatment of uLMS.

## Conflict of interest

The authors declare no conflict of interest.

## Author contributions

JG‐M and JWC conceived the project. JG‐M, PH and OG conducted the *in vitro* experiments and analysed the data. JG‐M, RMF and GK processed human tissue samples and performed gene expression data analyses. VZ analysed tissue mitoses. All authors interpreted the data. TBP, KL, and JWC supervised the study. JG‐M wrote the manuscript with contributions from all authors.

### Peer review

The peer review history for this article is available at https://www.webofscience.com/api/gateway/wos/peer‐review/10.1002/1878‐0261.13440.

## Supporting information


**Fig. S1.** Example of tumour tissue with tumour‐adjacent myometrium tissue.
**Fig. S2.** Presence of metastasis according to collagen features of primary tumours.
**Fig. S3.** MMP14 activity regulates uterine leiomyosarcoma cell proliferation.
**Fig. S4.** YAP activity regulates uterine leiomyosarcoma cell proliferation.
**Fig. S5.** Mevalonate pathway gene expression is enhanced in uterine leiomyosarcoma.

**Table S1.** Details of the tissue patient cohort.

**Table S2.** Differentially expressed genes between uLMS tumours with high and low collagen density.

**Data S1.** Figure legends.

## Data Availability

All data needed to evaluate the conclusions in the paper are present in the paper and/or the Supplementary Materials. Additional data related to this paper may be requested from the authors.
