# Supplementary material for: MMP14 expression and collagen remodelling support uterine leiomyosarcoma aggressiveness
Source: Mol Oncol. 2023 Apr 28;18(4):850–65. doi: 10.1002/1878-0261.13440 (PMC10994236; doi:10.1002/1878-0261.13440)

Supplemnetary Figures

MMP14 expression and collagen remodelling support uterine leiomyosarcoma aggressiveness

Jordi Gonzalez-Molina , Paula Hahn, Raul Maia Falcão , Okan Gultekin , Georgia Kokaraki ,
Valentina Zanfagnin, Tirzah Braz Petta , Kaisa Lehti , Joseph W. Carlson

Supplementary Fig. 1

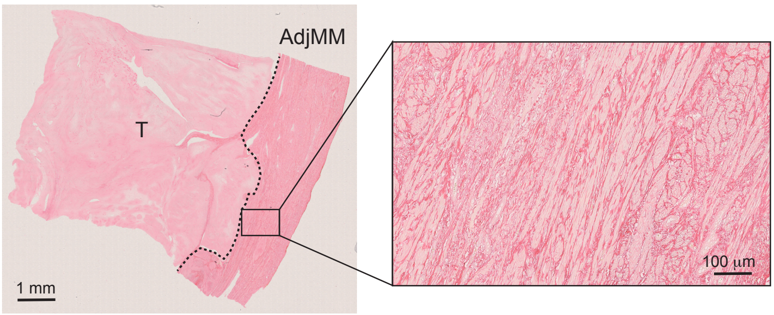

Supplementary Fig. 2

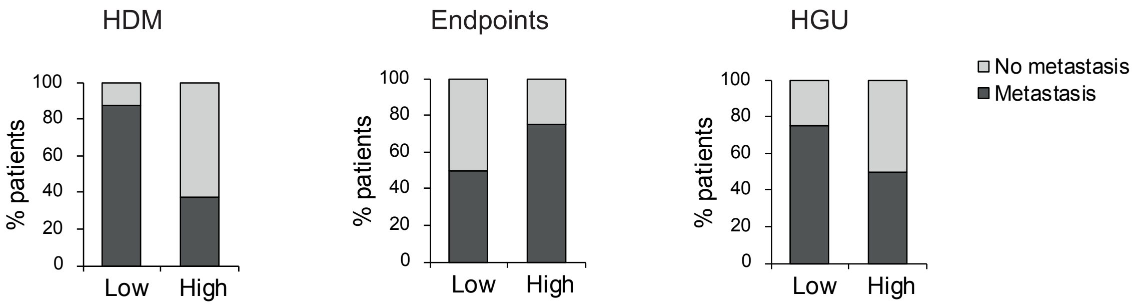

Supplementary Fig. 3

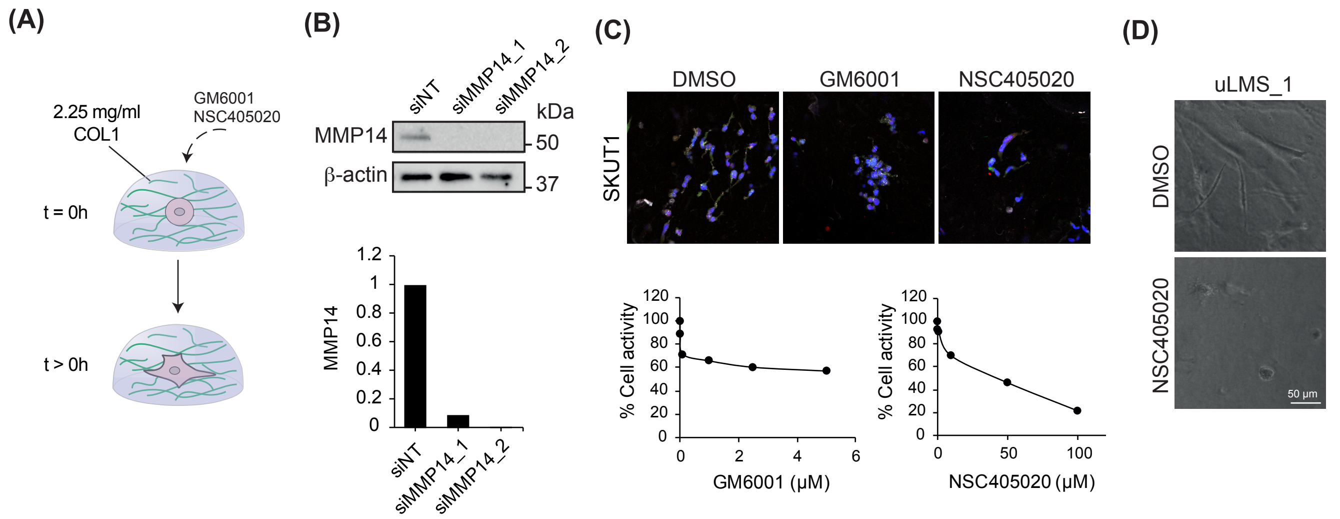

Supplementary Fig. 4

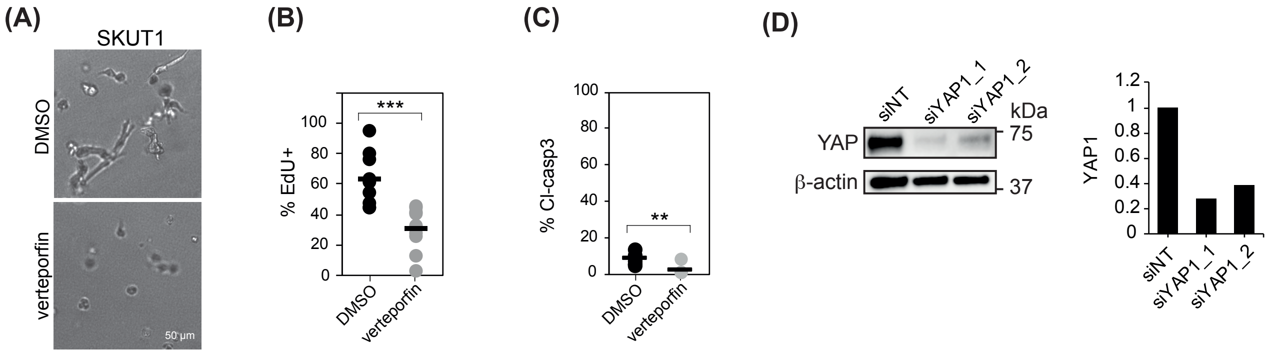

Supplementary Fig. 5

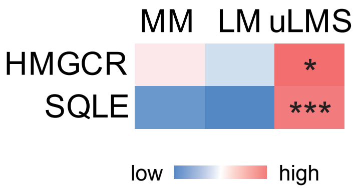

Supplement: Supplementary file 1 — Fig. S1. Example of tumour tissue with tumour‐adjacent myometrium tissue. Fig. S2. Presence of metastasis according to collagen features of primary tumours. Fig. S3. MMP14 activity regulates uterine leiomyosarcoma cell proliferation. Fig. S4. YAP activity regulates uterine leiomyosarcoma cell proliferation. Fig. S5. Mevalonate pathway gene expression is enhanced in uterine leiomyosarcoma. [file MOL2-18-850-s002.pdf]
