# Supplementary material for: MMP14 expression and collagen remodelling support uterine leiomyosarcoma aggressiveness
Source: Mol Oncol. 2023 Apr 28;18(4):850–65. doi: 10.1002/1878-0261.13440 (PMC10994236; doi:10.1002/1878-0261.13440)
Supplement: Supplementary file 4 — Data S1. Figure legends. [file MOL2-18-850-s001.docx]

**Supplementary Figure legends**

**Supplementary Fig. 1**. Example of tumour tissue with tumour-adjacent myometrium tissue. Example of tumour tissue (T) with tumour-adjacent myometrium tissue (AdjMM) stained for collagen with picrosirius red showing aligned collagen fibres in AdjMM. Left scale bar indicates 1 mm and the right scale bar indicates 100 μm.

**Supplementary Fig 2**. Presence of metastasis according to collagen features of primary tumours. Percentage of patients presenting metastasis at the time the primary tissue was resected in uLMS patients with high (n = 8) and low (n = 8) high-density matrix (HDM), fibre End points, and hyphal growth unit (HGU).

**Supplementary Fig 3**. MMP14 activity regulates uterine leiomyosarcoma cell proliferation. **(A)**, Schematic representation of the 3D collagen-based models used to embed cells in. **(B)** Representative western blot (top) and quantification (bottom) showing MMP14 protein expression is reduced upon siRNA knockdown in SKUT1 cells. **(C)** Representative images (top) and quantification (bottom) of the response of SKUT1 cells to pan-MMP inhibition (GM6001) or the specific inhibition of MMP14 (NSC405020) based on total ATP content. Each data point indicates the average of 3 technical replicates. Scale bar indicates 50 μm. **(D)** Representative images of patient-derived uLMS cells (scale bar indicates 50 μm) upon treatment with NSC405020.

**Supplementary Fig 4**. YAP activity regulates uterine leiomyosarcoma cell proliferation. **(A)** Representative examples of SKUT1 cells embedded in 3D collagen matrices and treated with YAP inhibitor verteporfin for 48 h. Scale bar indicates 50 μm. **(B)** Quantification of EdU incorporation, indicating cell proliferation rate, of cells from (**A**) (n = 9). **(C)** Quantification of apoptosis indicated by cleaved caspase 3 positivity (cl-casp3) of cells from (**A**). **(D)** Representative western blot (top) and quantification (bottom) showing YAP protein expression is reduced upon siRNA knockdown in SKUT1 cells.

**Supplementary Fig 5**. Mevalonate pathway gene expression is enhanced in uterine leiomyosarcoma. Expression of the genes of the mevalonate pathway HMGCR and SQLE in normal myometrium (MM; n = 31), leiomyoma (LM; n = 28), and uterine leiomyosarcoma (uLMS; n = 17) showing overexpression in uLMS. * p > 0.05, *** p > 0.001.
